# Public Preferences for Digital Health Data Sharing: Discrete Choice Experiment Study in 12 European Countries

**DOI:** 10.2196/47066

**Published:** 2023-11-23

**Authors:** Roberta Biasiotto, Jennifer Viberg Johansson, Melaku Birhanu Alemu, Virginia Romano, Heidi Beate Bentzen, Jane Kaye, Mirko Ancillotti, Johanna Maria Catharina Blom, Gauthier Chassang, Dara Hallinan, Guðbjörg Andrea Jónsdóttir, Aníbal Monasterio Astobiza, Emmanuelle Rial-Sebbag, David Rodríguez-Arias, Nisha Shah, Lea Skovgaard, Ciara Staunton, Katharina Tschigg, Jorien Veldwijk, Deborah Mascalzoni

**Affiliations:** 1 Institute for Biomedicine (Affiliated Institute of the University of Lübeck) Eurac Research Bolzano Italy; 2 Department of Biomedical, Metabolic and Neural Sciences University of Modena and Reggio Emilia Modena Italy; 3 Centre for Research Ethics and Bioethics Department of Public Health and Caring Sciences Uppsala University Uppsala Sweden; 4 Curtin School of Population Health Curtin University Bentley Australia; 5 Department of Health Systems and Policy University of Gondar Gondar Ethiopia; 6 Centre for Medical Ethics Faculty of Medicine University of Oslo Oslo Norway; 7 Norwegian Research Center for Computers and Law Faculty of Law University of Oslo Oslo Norway; 8 Centre for Health, Law and Emerging Technologies (HeLEX) Faculty of Law University of Oxford Oxford United Kingdom; 9 Centre for Health, Law and Emerging Technologies Melbourne Law School University of Melbourne Melbourne Australia; 10 Center for Neuroscience and Neurotechnology University of Modena and Reggio Emilia Modena Italy; 11 Ethics and Biosciences Platform (Genotoul Societal), Genotoul Centre for Epidemiology and Research in Population Health, UMR1295 Inserm Toulouse France; 12 Centre for Epidemiology and Research in Population Health National Institute for Health and Medical Research (Inserm)/Toulouse University Toulouse France; 13 FIZ Karlsruhe – Leibniz-Institut für Informationsinfrastruktur Eggenstein-Leopoldshafen Germany; 14 Social Science Research Institute University of Iceland Reykjavik Iceland; 15 Departamento de Filosofía I Universidad de Granada Granada Spain; 16 FiloLab-UGR Department of Philosophy 1 University of Granada Granada Spain; 17 Centre for Medical STS (MeST) Department of Public Health University of Copenhagen Copenhagen Denmark; 18 School of Law University of Kwazulunatal Durban South Africa; 19 Department of Cellular, Computational, and Integrative Biology University of Trento Trento Italy; 20 Erasmus School of Health Policy & Management Erasmus University Rotterdam Rotterdam Netherlands; 21 Erasmus Choice Modeling Centre Erasmus University Rotterdam Rotterdam Netherlands

**Keywords:** governance, digital health data, preferences, Europe, discrete choice experiment, data use, data sharing, secondary use of data

## Abstract

**Background:**

With new technologies, health data can be collected in a variety of different clinical, research, and public health contexts, and then can be used for a range of new purposes. Establishing the public’s views about digital health data sharing is essential for policy makers to develop effective harmonization initiatives for digital health data governance at the European level.

**Objective:**

This study investigated public preferences for digital health data sharing.

**Methods:**

A discrete choice experiment survey was administered to a sample of European residents in 12 European countries (Austria, Denmark, France, Germany, Iceland, Ireland, Italy, the Netherlands, Norway, Spain, Sweden, and the United Kingdom) from August 2020 to August 2021. Respondents answered whether hypothetical situations of data sharing were acceptable for them. Each hypothetical scenario was defined by 5 attributes (“data collector,” “data user,” “reason for data use,” “information on data sharing and consent,” and “availability of review process”), which had 3 to 4 attribute levels each. A latent class model was run across the whole data set and separately for different European regions (Northern, Central, and Southern Europe). Attribute relative importance was calculated for each latent class’s pooled and regional data sets.

**Results:**

A total of 5015 completed surveys were analyzed. In general, the most important attribute for respondents was the availability of information and consent during health data sharing. In the latent class model, 4 classes of preference patterns were identified. While respondents in 2 classes strongly expressed their preferences for data sharing with opposing positions, respondents in the other 2 classes preferred not to share their data, but attribute levels of the situation could have had an impact on their preferences. Respondents generally found the following to be the most acceptable: a national authority or academic research project as the data user; being informed and asked to consent; and a review process for data transfer and use, or transfer only. On the other hand, collection of their data by a technological company and data use for commercial communication were the least acceptable. There was preference heterogeneity across Europe and within European regions.

**Conclusions:**

This study showed the importance of transparency in data use and oversight of health-related data sharing for European respondents. Regional and intraregional preference heterogeneity for “data collector,” “data user,” “reason,” “type of consent,” and “review” calls for governance solutions that would grant data subjects the ability to control their digital health data being shared within different contexts. These results suggest that the use of data without consent will demand weighty and exceptional reasons. An interactive and dynamic informed consent model combined with oversight mechanisms may be a solution for policy initiatives aiming to harmonize health data use across Europe.

## Introduction

The increasing growth and relevance of digital tools and approaches for health [1] meant that developing European governance for digital health data has become a compelling task [2]. There are many competing initiatives (eg, public, private, and those initiated by the European Union [EU]) to facilitate cross-border data sharing. The European Commission has developed legislation to enable the sharing of health data within Europe, such as the sharing of public sector data under the Data Governance Act [3] and the Data Act [4], as well as the proposed draft European Health Data Space (EHDS) [5]. The draft EHDS seeks to harmonize the processes for accessing and sharing electronic health data across EU Member States for certain secondary purposes by fostering common standards and requirements. Such initiatives will have an impact on millions of people’s lives across Europe, and the development of necessary infrastructure will involve considerable investment of resources. Empirical studies are needed to understand the differences and commonalities in the views of a wide range of publics on such sharing of data for secondary purposes and where the tensions between views might lie. This will ensure that such initiatives have full public support and are deemed to be trustworthy, but also that they can be designed to meet and engage with a wide range of public expectations about the use of data [6,7].

Review studies investigating public views on health data sharing have mapped overarching issues, ethical values, conditions, and factors that affect people’s willingness to share health data [6,8-11]. The reviews examined studies conducted in several European countries, the United States, Canada, Japan, Australia, and New Zealand. Most of these reviews showed generalized but conditional public support for health data sharing. Expected contribution to the common good, preserving data security, and transparency on data use were among the conditions for public support of data sharing [6,8,10]. Concerns about privacy breaches, data security, data management, and misuse or abuse of data, and a general hesitancy toward commercial purposes with data use were found. On the other hand, these reviews also found a variance in views and attitudes among the studies, which, according to the authors, affected the comparability and generalizability of the findings of the studies. This variance was interpreted to be the result of (1) sociocultural and geographical factors [6,8,10]; (2) diverse and specific study populations [6]; (3) underrepresentation of specific age groups [6]; (4) differences in the perception of sensitivity of data [6]; and (5) differing methodological approaches [8,10].

In our analysis of the empirical literature on privacy research, we noticed 2 main issues. First, there is a paucity of research on peoples’ preferences for the governance of health data sharing by a variety of users and how that varies between settings. The context of data sharing explored in these studies was often within the research or public health context and did not consider the movement of data between public and commercial sectors. Second, although much work has been conducted using a variety of qualitative and quantitative methods, there are only a few preference studies based on discrete choice experiments (DCEs). DCEs aim to elicit and understand respondents’ preferences and trade-offs in decision-making. Studies that adopted this method focused on data-sharing preferences within research [12,13] or the public health context [14]. Only 1 study in countries in Northern Europe investigated preferences for digital health data sharing in different contexts by including a variety of collectors, users, reasons, information and consent, and extents of review [15]. The use of a DCE approach allowed the authors to explore how people balance different aspects when contexts change and how people make trade-offs in verisimilar situations of data-sharing decisions. This study showed shared preferences for being informed with the possibility of opting out. However, national differences in preferences for the review process oversighting data sharing and for the reason of data re-use were evident [15]. To foster evidence-based policies on health data sharing and better understand preferences on the use of digital health data across a wider group of European countries, we built upon the DCE study in Northern European countries and conducted a large-scale investigation of European public preferences.

## Methods

### Details of DCE

DCEs can be used to quantify preferences for products or services and are increasingly used within the health care setting [16,17]. This method is based on the Random Utility Theory (RUT) and requires respondents to answer several choice tasks. Such tasks present two or more profiles of a specific good or service. The profiles are described based on their characteristics (ie, attributes) with varying levels [18-20]. Within each choice task, respondents are asked to choose the profile with the highest personal utility [21-24]. Based on the choices respondents make, the impact of each attribute on the total utility is estimated, and the relative importance of the included attributes can be inferred [24-26]. The DCE developed as part of this study followed the guidelines of good research practice [27].

### Recruitment and Data Collection

This study extends a previous DCE [15]. Recruitment and data collection in the previous study have been described [15]. The survey was extended to the following countries (in alphabetical order): Austria, Denmark, France, Germany, Ireland, Italy, the Netherlands, and Spain. Within each of these countries, a representative sample of 400 members of the general public was used. Respondents were recruited via a recruitment service, SurveyEngine [28], and received compensation according to customary agreements between SurveyEngine and the participant. Each national sample was representative (in composition according to gender and age) of the general population of each country. In order to achieve that, we provided the recruiting company with percentage data about the sample composition for age (18-39 years old, 40-59 years old, and ≥60 years old) and gender (female and male; % for each age group) based on the most recent (at the time of sample design) national statistics data available for each country. With these data, the recruiting company recruited participants of different ages and genders to obtain a sample with a defined number of respondents (ie, 400 respondents for the survey and 40 respondents for the ranking exercise) for meeting the requested composition. Data were collected from August to November 2020 for the original study, and from April to August 2021 for the other 8 countries.

### Ethical Considerations

This study was conducted in accordance with national and international laws and regulations regarding the protection of personal information, privacy, and human rights. Before starting the survey, prospective participants were informed about research participation (study aim, possibility of withdrawing from survey participation, possibility of withdrawing consent to the use of data for research until the data are analyzed, risks and benefits, and data processing). Participation was voluntary. Respondents provided consent to the use of the collected data for research purposes. Data collected by the recruitment service SurveyEngine were encoded. For data analysis, researchers accessed deidentified data.

Ethics approvals for the original study have been described previously [15]. For the extension of the study, the Ethics Committee of the Erasmus School of Health Policy and Management from the University (reference number 21-011) waived the necessity of formal testing by a medical ethics committee.

### Selection of Attributes and Levels

A 4-step approach was used for this process: (1) literature review; (2) 14 focus group discussions (including a nominal group technique [29] with members of the general population in the United Kingdom, Sweden, and Iceland); (3) expert interviews; and (4) confirmation ranking surveys. Steps 1 to 3 were part of a previous study described in detail elsewhere [15,30]. As a fourth step, an online ranking exercise was formulated and conducted in each of the additional 8 countries of interest to confirm the selected attributes and levels for the DCE. Respondents ranked a set of 12 items, which included the attributes and levels from step 2 listed above. This online ranking survey was sent to a representative sample of 40 respondents in each country. Recruitment and data collection for the ranking exercise were conducted from April to August 2021 by SurveyEngine. In all countries, the attributes included in the previous studies were among the highest ranked attributes across countries. The final attributes and levels are described in Table 1.

**Table 1:** Attributes and levels included in the discrete choice experiment.

Attribute and level		Description
**Data collector**		
	Health care provider	Your health care provider (hospital or general practitioner) who collected health information about your care.
	Technological company	A technological company with which you used a service, program, or application for a phone or computer.
	Academic research project	An academic research project where you participated and health information about you was collected.
**Data user**		
	National authority	A national authority like the public health authority or information and commissioner’s office, which is responsible for the population’s health.
	Technological company	A technological company that develops health applications, which can be used to predict diagnosis.
	Pharmaceutical company	A pharmaceutical company that develops and manufactures new medicines.
	Academic research project	An academic research project that produces new knowledge by testing hypotheses and theories about human health.
**Reason for data use**		
	Quality evaluation	Evaluate the quality of the data user’s product or service and plan for resource allocation.
	Development of a new product or service	Develop new products or services, such as medical devices, drugs, or applications for phones, or new health services or programs.
	Promotion, advertising, and marketing	Promoting, advertising, and marketing products or services to personalize communications.
	Investigating policy initiative	Investigating policy initiatives at the national level. The investigation could improve services for a specific part of the population or identify new measures to improve public health.
**Information on data sharing and consent**		
	Informed and consent	Informed and asked to consent that health information about you is being shared and used in a new context.
	Not informed	Not informed that health information about you is being shared and used in a new context.
	Informed	Informed that health information is being shared and used in a new context.
	Informed and ability to opt-out	Informed that health information is being shared and used in a new context and you are offered to opt-out.
**Availability of review process**		
	Review of transfer and use	A committee will review the transfer and use of your health information in a new context.
	No review	No review of data sharing.
	Review of transfer	A committee will review the transfer of your health information to a new context.

### Experimental Design, Survey Construction, and Pilot Testing

**Figure 1:**
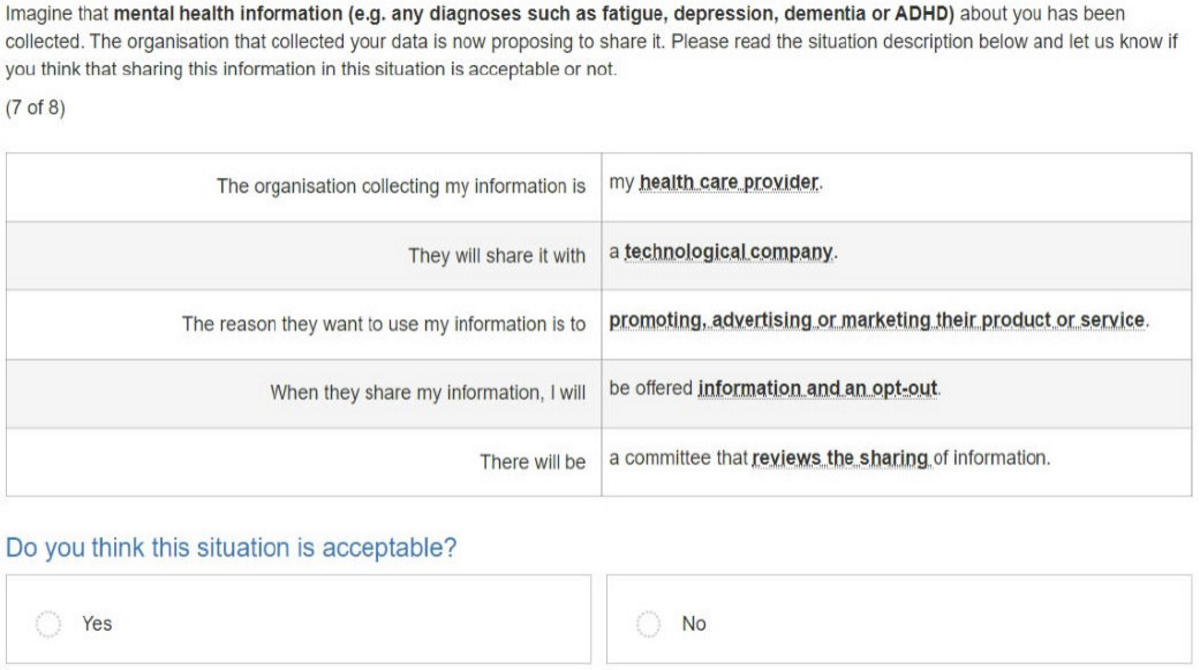
Example of a choice task used in this study.

A Bayesian D-efficient design using 500 Halton draws and 1000 repetitions was constructed for this DCE, which was developed using NGene (version 1.2.1; ChoiceMetrics). Best guess estimates were used as prior information for the initial design. No interactions between attributes were included, and level balance was optimized. A total of 32 unique choice tasks were generated, which were divided over 4 blocks of 8 choice tasks. Respondents were randomized to a block and 2 situations of “type of information” (see below). Each choice task consisted of 1 profile representing a data sharing situation, and respondents could either accept or reject their data being shared in such a situation (Figure 1).

The online survey had 3 sections. The first part contained questions regarding demographic characteristics (eg, age, gender, nationality, and educational level), as well as questions on health-related professions, previous participation in research, use of digital technologies for health, and health status. The second part included the DCE. Each respondent was confronted with 8 choice tasks from 2 types of health information (ie, lifestyle information, physical health, mental health, or genetic information). This resulted in 16 choice tasks for each respondent. Prior to answering the choice tasks, respondents received detailed information on the type of health information that was applicable to the choice task and the meaning of all attributes and levels. Next, respondents were presented with an example choice task. The third part of the questionnaire measured trust and included related attitudinal questions.

The survey was further developed with 8 think-aloud interviews with members of the general public in the United Kingdom, Sweden, and Iceland, and a 2-day workshop with external experts (areas of expertise included law, philosophy, ethics, social science, and stated preference research) [15,30]. The survey was pilot tested in all countries (n=50 in the United Kingdom, Iceland, Sweden, and Norway, and n=40 in the other countries). Data gathered from the pilots of the previous study were analyzed, and attribute level estimates were used as prior input for the final DCE design. Pilot tests for this study were performed approximately 2 weeks before the data collection on a large scale in each country.

The survey and the ranking exercise were translated from English into the respective national language of each country included in the study.

### Statistical Analysis

For the statistical analysis, the countries were categorized into regions within Europe according to the United Nations classification. Northern Europe includes Sweden, Norway, the United Kingdom, Iceland, Ireland, and Denmark. Central Europe includes the Western European countries of the Netherlands, Germany, France, and Austria. Southern Europe includes Italy and Spain.

Descriptive statistics (means and frequencies) were presented for each country and region. Chi-square tests and 1-way ANOVA were conducted to examine differences in demographics across regions and across countries within those regions. Results were considered statistically significant at *P*<.05.

Panel latent class models (LCMs) were applied to determine attribute level estimates [31]. Separate models were conducted on the full pooled data set and within each of the 3 European regions considered. Such models account for the multilevel structure of the data and detect preference heterogeneity [32]. All attributes were considered nonlinear and were therefore effect-coded [24,33]. Based on model fit tests (Akaike information criteria and log likelihood), the model most suitable for our data was selected (models ranging from 1 to 6 classes were tested) [34]. The final utility equation is as follows:

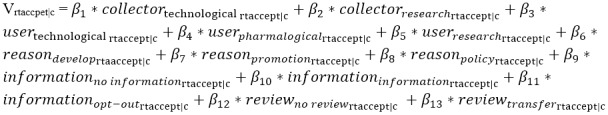

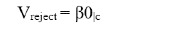





The systematic utility component (V) describes the observable utility that participant “r” belonging to class “c” reported for alternative “a” in choice task “t.” β_0_ represents the alternative specific constant for rejecting to share information, and β_1_-β_13_ are the attribute level estimates. A positive and significant value means that respondents prefer to reject data sharing, and a negative and significant value implies that respondents favor data sharing.

Regarding the interpretation of the model results, a positive and significant coefficient shows that the corresponding attribute level is preferred by respondents or provides utility compared to the reference. In contrast, a negative and significant coefficient indicates that the corresponding attribute level is not preferred or provides disutility compared to the reference.

In addition to the above-specified utility function, a class assignment model was fitted to each of the LCMs. In an LCM, the respondent sample is divided into subgroups (classes) based on the expressed preferences. Each class shares a pattern of preferences. Class share indicates the proportion of respondents (%) within each specific class. Within the latent class analysis (LCA) on the full pooled data set, variables identifying the European region to which the individual countries belong were added, and in the LCAs conducted for the regional data, a variable representing the country to which the data belongs was included. A positive and significant coefficient indicates that the respondents from that specific country are more likely to belong to that specific class compared to the country used as a reference. On the contrary, a negative and significant coefficient indicates that the respondents from that specific country are less likely to belong to that specific class.

#### Attribute Relative Importance

The difference between the highest and lowest attribute level estimate was calculated for each attribute. The largest difference value received a score of 1, representing the attribute that was deemed most important by participants. The other difference values were divided by the largest difference value, resulting in a relative distance between all other attributes and the most important attribute.

Relative importance scores for the attributes relative to the most important attribute were calculated based on the results of the LCMs, separately for all classes. The class-adjusted relative importance was calculated by computing the relative importance score of all attributes in each class separately, as described above, after which they were weighted according to class assignment probability.

## Results

### Respondents’ Characteristics

A total of 5321 completed surveys were obtained. Due to a short completion time (less than 5 minutes), 306 completed surveys were excluded, and thus, 5015 completed surveys were included in the analyses (see Table 2 for a full overview of the demographics). The mean age of respondents across countries was 49.75 years.

The mean age and educational level of respondents differed significantly among regions. The highest mean age was in Central Europe. In comparison with Central and Southern Europe, there was a greater percentage of respondents with higher education in Northern Europe.

**Table 2:** Sociodemographic characteristics of respondents in each country and across regions.

Variable		Sample size, n	Age (years)^a^	Gender, %			Education, %^a^
			Value, mean (SD)	Female	Male	Higher^b^	
**Northern Europe**		2754	49.10 (16.86)^a^	51.7^c^	48.3^c^	46.1^a^	
	Sweden	492	50.19 (16.96)	52.2	47.8	35.2	
	Norway	477	48.40 (17.26)	51.8	48.2	38.3	
	United Kingdom	441	49.36 (16.06)	52.2	47.8	51.1	
	Iceland	542	48.32 (17.20)	50.0	50.0	56.9	
	Ireland	403	47.91 (15.83)	52.1	47.9	55.0	
	Denmark	399	50.54 (17.45)	52.6	47.4	39.2	
**Central Europe**		1564	50.96 (15.96)^a^	51.4	48.6	45.4^a^	
	Netherlands	361	52.52 (16.26)	51.2	48.8	43.3	
	France	396	50.70 (15.92)	51.8	48.2	38.4	
	Germany	400	51.33 (16.02)	51.0	49.0	50.9	
	Austria	407	49.48 (15.52)	51.6	48.4	49.0	
**Southern Europe**		697	49.60 (14.35)^a^	52.7	47.3	42.3^a^	
	Spain	368	48.55 (14.09)	52.2	47.8	46.5	
	Italy	329	50.77 (14.54)	53.2	46.8	37.7	

^a^*P*<.01; chi-square or 1-way ANOVA significance level among regions or within regions.

^b^Higher education corresponds to university-level education (bachelor and postgraduate).

^c^*P*<.05; chi-square or 1-way ANOVA significance level among regions or within regions.

### Preferences for Digital Health Data Sharing in Europe

A 4-class LCM was fitted (Table 3). While class 1 respondents strongly a priori preferred not to share their health data, class 3 respondents were very positive toward data sharing. Class 2 and 4 respondents a priori preferred not to share their health data, but their preference could be heavily impacted by attribute levels of the situation. Respondents generally showed a disutility for a technological company collecting their data over their health care provider or an academic research project. They also, on average (except for class 2), preferred a national authority or academic research project to use their data over a technological or pharmaceutical company. Across all classes, respondents reported a strong disutility for using their data for promotion, advertising, and marketing over developing a new product or service, investigating a policy initiative, and evaluating quality. Respondents preferred being informed and asked to provide their consent over being informed and having the possibility to opt-out, only informed, and not informed. Respondents preferred the review of data transfer and use, or the review of the transfer over the absence of a review.

**Figure 2:**
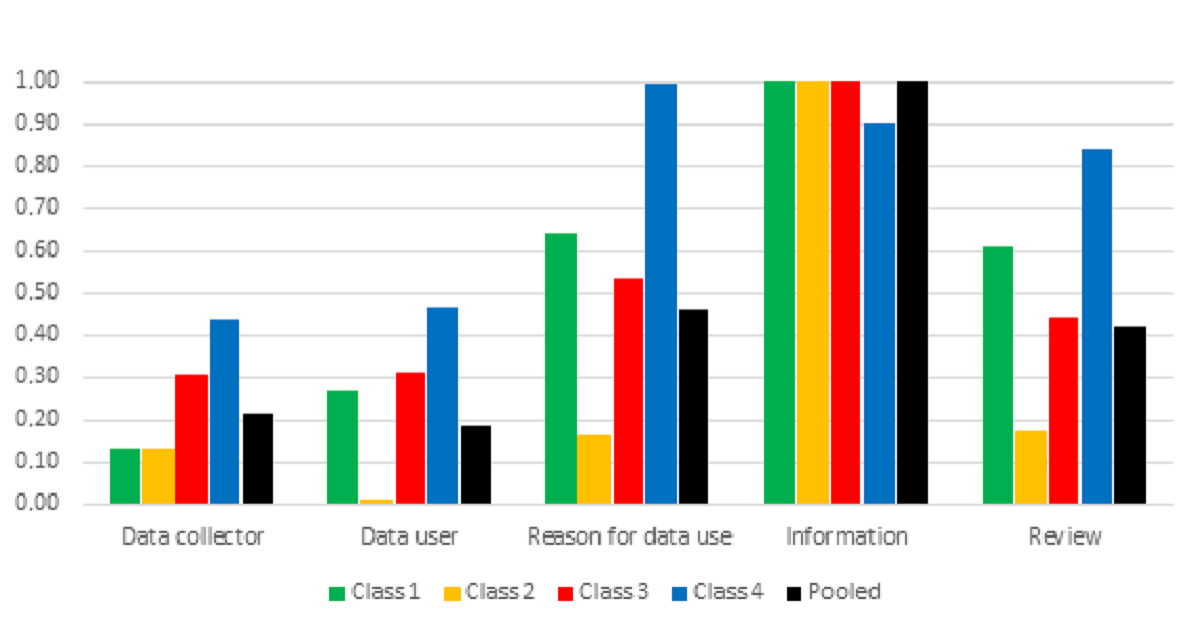
Relative importance of attributes for the latent classes identified through the latent class model with data from all the countries.

The most important attribute for the whole sample was “information on data sharing and consent,” followed by “availability of review process,” “reason for data use,” “data user,” and “data collector.” Moreover, “information on data sharing and consent“ was the most important attribute in all latent classes, except in class 4, where “reason for data use” was the most important attribute. While the other attributes seemed relatively unimportant for respondents in class 2, the opposite was found for respondents in class 4 (Figure 2).

**Table 3:** Latent class model with data from all the countries.

Attribute and level			Class 1^a^					Class 2^a^					Class 3^a^					Class 4^a^		
			Coefficient		SE		Coefficient			SE		Coefficient			SE		Coefficient			SE
**Data collector**																				
	Health care provider (reference)	0.03		0.06		0.20^b^			0.04		0.35^b^			0.05		0.21^b^			0.02	
	Technological company	−0.13^c^		0.06		−0.43^b^			0.04		−0.40^b^			0.05		−0.31^b^			0.02	
	Academic research project	0.10^d^		0.06		0.23^b^			0.04		0.05			0.05		0.10^b^			0.02	
**Data user**																				
	National authority (reference)	0.15^c^		0.08		−0.01			0.04		0.28^b^			0.07		0.16^b^			0.02	
	Technological company	−0.33^b^		0.09		0.00			0.05		−0.40^b^			0.06		−0.34^b^			0.02	
	Pharmaceutical company	0.01		0.08		−0.02			0.05		−0.23^b^			0.07		−0.04^d^			0.02	
	Academic research project	0.17^c^		0.08		0.03			0.04		0.35^b^			0.07		0.22^b^			0.02	
**Reason for data use**																				
	Quality evaluation (reference)	0.18^c^		0.08		0.43^b^			0.05		0.44^b^			0.06		0.41^b^			0.02	
	Development of a new product or service	0.49^b^		0.09		−0.11^c^			0.05		0.33^b^			0.06		0.24^b^			0.02	
	Promotion, advertising, marketing	−0.66^b^		0.10		−0.42^b^			0.06		−0.87^b^			0.08		−0.78^b^			0.03	
	Investigating policy initiative	−0.02		0.08		0.10^c^			0.05		0.10			0.06		0.13^b^			0.02	
**Information and consent**																				
	Informed and consent (reference)	0.58^b^		0.10		2.04^b^			0.09		0.99^b^			0.08		0.35^b^			0.03	
	Not informed	−1.22^b^		0.10		−3.10^b^			0.08		−1.45^b^			0.09		−0.72^b^			0.04	
	Informed	0.14		0.10		−0.44^b^			0.05		−0.10			0.07		0.03			0.03	
	Informed and opt-out	0.50^b^		0.10		1.50^b^			0.06		0.56^b^			0.07		0.34^b^			0.03	
**Review**																				
	Review of transfer and use (reference)	0.16^c^		0.07		0.30^b^			0.05		0.32^b^			0.05		0.34^b^			0.02	
	No review	−0.63^b^		0.07		−0.59^b^			0.05		−0.70^b^			0.06		−0.66^b^			0.02	
	Review of transfer	0.47^b^		0.07		0.29^b^			0.04		0.37^b^			0.05		0.31^b^			0.02	
Rejecting data sharing (intercept)			3.19^b^		0.08		0.86^b^			0.05		−2.24^b^			0.08		0.17^b^			0.03
**Class membership variables^e^**																				
	Constant	−0.38^b^		0.06		−0.52^b^			0.08		−0.87^b^			0.09		Reference			N/A^f^	
	Central Europe	−0.65^b^		0.10		−0.09			0.10		0.20^c^			0.10		Reference			N/A	
	Southern Europe	0.11		0.12		0.38^b^			0.12		−0.66^b^			0.18		Reference			N/A	

^a^The class share is as follows: Class 1, 22.04%; Class 2, 23.61%; Class 3, 15.7%; Class 4, 38.66%.

^b^Significant at the 1% level.

^c^Significant at the 5% level.

^d^Significant at the 10% level.

^e^Reference class membership: Northern Europe.

^f^N/A: not applicable.

As a data collector, class 1 and 2 respondents preferred an academic research project over their health care provider (not significant in class 1). The opposite pattern was found in classes 3 and 4. In all the classes, respondents expressed disutility for a technological company compared to their health care provider.

Compared to sharing data with a national authority, class 1, 3, and 4 respondents preferred to share data with an academic research project and showed disutility for a technological company. Class 3 and 4 respondents also showed disutility for a pharmaceutical company compared to a national authority, even though this was more acceptable than a technological company. In class 2, this attribute did not impact decision-making.

Health data sharing for promotion, advertising, and marketing purposes provided the most disutility in all classes. For class 1 respondents, developing a new product or service was preferred over quality evaluation. Sharing digital health data for quality evaluation was most acceptable by class 2, 3, and 4 respondents. For class 2 respondents, this was followed by investigating a policy initiative, while developing a new product showed disutility. For class 3 and 4 respondents, developing a new product or service was less acceptable than quality evaluation. For class 4 respondents, investigating a policy initiative was also acceptable but less than quality evaluation.

Furthermore, respondents preferred being informed and providing consent for data sharing over being informed and being offered to opt-out. Class 2 respondents also expressed disutility for being only informed. Sharing health data without being informed resulted in substantial disutility in all classes.

Class 1 and 3 respondents preferred review of data transfer only compared to review of data transfer and use. The opposite was found for class 2 and 4 respondents. The absence of review provided disutility in all the classes.

Compared to respondents from Northern Europe, respondents from Central Europe were more likely to belong to class 3 and less likely to belong to class 1 (compared to class 4). Respondents from Southern Europe were more likely to belong to class 2 and less likely to belong to class 3.

### Regional Preference and Regional Heterogeneity in Europe

A 4-class latent model was fitted to the northern, central, and southern regions showing preference heterogeneity for sharing health data digitally within each region (see Tables S1-S3 in Multimedia Appendix 1).

**Figure 3:**
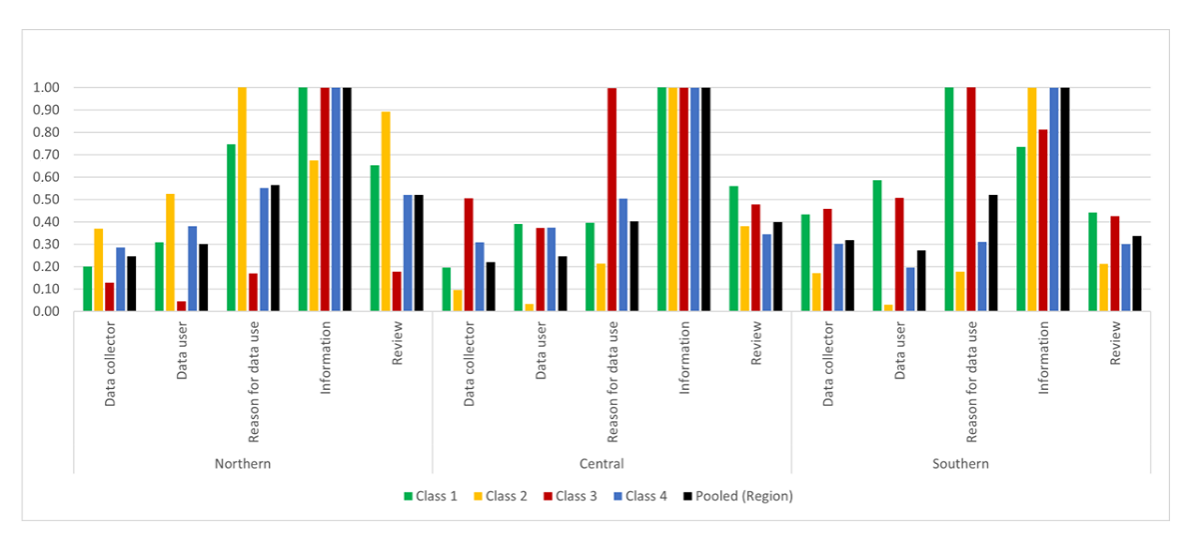
Relative importance of attributes for the latent classes of Northern, Central, and Southern Europe. Classes are ordered according to respondents’ a priori preference for sharing their health data, with class 1 as the most negative toward data sharing, class 4 as the most positive, and classes 2 and 3 as showing conditional support or indifference (see Multimedia Appendix 1).

In each regional sample considered as a whole, “information on data sharing and consent” was the most important attribute. This was followed by “availability of review process” or “reason for data use,” while “data user” or “data collector” was the least important attribute (Figure 3). Within each region, “information on data sharing and consent” was the most important attribute for most classes in Europe. Within each region, there was a class for which the other attributes were relatively unimportant compared to the most important attribute (class 3 for Northern Europe, class 2 for Central Europe, and class 2 for Southern Europe).

In Northern Europe, “information on data sharing and consent” was the most important attribute in all classes, except in class 2, where “reason for data use” was the most important, and “availability of review process” was relatively more important compared to the other classes.

Within Central Europe, a similar pattern was shown; however, for class 3, “information on data sharing and consent” and “reason for data use” were relatively equally important attributes. In 2 classes of Central Europe, “availability of review process” had less importance compared to “data collector” (for class 3 respondents) or “data user” (for class 4 respondents).

Finally, in Southern Europe, “information on data sharing and consent” was the most important attribute in classes 2 and 4, while “reason for data use” was the most important attribute in classes 1 and 3, where “availability of review process” was less important than “data user” or “data collector.”

## Discussion

### Principal Findings and Comparison With Prior Work

The results showed that people in the European countries sampled shared, to a certain degree, general commonalities regarding what is important to them about digital health data governance. However, they also showed different priorities and preferences depending on their region of residence.

#### Importance of Information on Digital Health Data Sharing

In general, “information on data sharing and consent” was the most important attribute when considering the whole respondent population and the pooled regional subgroups. “Information on data sharing and consent” was the most important attribute for 61.35% of European respondents (Table 3), 67.37% of Northern European respondents (Table S1 in Multimedia Appendix 1), all Central European respondents (Table S2 in Multimedia Appendix 1), and 39.62% of Southern European respondents (Table S3 in Multimedia Appendix 1). Furthermore, for a subgroup of respondents in each region, the other attributes were relatively unimportant, thus reinforcing the primary relevance of information and consent for data sharing in the preferences of European residents.

When asked about their preferences for receiving information and the possible mechanisms of consent, respondents generally found it most acceptable to be informed and asked to consent to data sharing. These findings indicated that it is valuable for respondents to exert control over digital health data sharing by being made aware of their data use and providing active consent to such use. Previous literature supports this finding, showing the high importance for individuals to have control over data sharing [35-37]. This suggests that to reflect broadly shared European values, establishing processes that guarantee access to transparent information on data sharing and provide mechanisms for citizens to express consent is crucial for governance initiatives at the European level.

Regional heterogeneity emerged in the information and consent preferences. For Southern European respondents, being informed was essential. However, a variety of preferences related to information and consent was expressed (Table S3 in Multimedia Appendix 1): active consent (39.62%), opt-out (35.12%), and only informed (25.27%). All Central European respondents preferred being informed and asked to consent, thus generally showing a high interest in controlling their data being shared and used. More than half of Northern European respondents (56.66%; Table S1 in Multimedia Appendix 1) preferred being informed and having the possibility to opt-out, while the rest preferred being informed and providing active consent. By including data from Ireland and Denmark in this study, the pattern of preferences for information and the type of consent expressed by Northern European respondents slightly changed compared to what was found in the previous study [15] conducted in Sweden, Norway, Iceland, and the United Kingdom, whose respondents preferred to be informed and have the possibility to opt-out.

In general, a review was also critical (second or third attribute as importance) for respondents. Throughout Europe and the regions, respondents consistently found the review of use and transfer, or the review of transfer only, most acceptable. They found the absence of a review process least acceptable (with 1 exception in Southern Europe, see below). This is in line with the results of previous studies, which showed that the presence of reviewing mechanisms and oversight institutions regulating the data-sharing process was important for people [35,38].

#### Reluctance to Use Digital Health Data by Private Entities

Overall, respondents found it the least acceptable for private enterprises to collect or use their digital health data. Specifically, European respondents generally found technological companies less acceptable as data collectors and users. A similar pattern was found in Northern Europe and Central Europe. Northern European respondents preferred the most when their health data were collected by their health care provider (75.47%) or an academic research project (24.53%) and used by a national authority (Table S1 in Multimedia Appendix 1). Central European respondents preferred the most their health care provider (81.49%) or an academic research project (18.51%) as a data collector and an academic research project as a data user (75.43%) (Table S2 in Multimedia Appendix 1). Most Southern European respondents (74.74%; Table S3 in Multimedia Appendix 1) preferred their data to be collected by their health care provider and found a technological company less acceptable as a data collector.

This may result from a differential level of trust in the public or private character of the entity collecting or using data. Previous studies showed that respondents generally found public institutions trustable, accountable, and pursuing the common good, while private companies were perceived in the opposite way [35,36]. We may speculate that people’s preferences are not only influenced by a perception or belief about who is most trustable, but it also has to do with legitimacy: believing that entities such as health care providers, national authorities, and academic institutions could legitimately collect and use their data, while others (such as technological companies) could not.

The overall dislike of technological companies as data collectors and users was accompanied by a general dislike of the use of data for commercial communication (marketing, promotion, and advertising). Marketing was previously found to be negatively perceived [37]. In general, quality evaluation or developing a new product or service were the most accepted purposes for data sharing. Furthermore, in Southern Europe, investigating a policy initiative was generally found to be less acceptable, while it was among the favorite purposes in Northern and Central Europe, perhaps indicating different levels of public trust in the perceived benefit of public health policy between Northern and Southern Europe.

The preferences of Southern European respondents were relatively more fragmented. Specifically, 1 subgroup of Southern Europeans (25.27%; Table S3 in Multimedia Appendix 1) showed a pattern of preferences that contrasted with the preferences expressed by the other respondents. This subgroup preferred private entities as data collectors (technological companies) and data users (pharmaceutical companies). They found it less acceptable for their health care provider to be a data collector, and an academic research project and a national authority to be a data user. They found the use of their data for policy development to be less acceptable, and they preferred to be only informed and preferred the absence of review mechanisms. It would be important to characterize this subgroup further and investigate the reasons for their expressed pattern of preferences.

Country-specific differences in preferences for health data governance have been previously reported [39-41]. The differences in preferences we found among respondents of different European regions may be related to general sociocultural and geopolitical factors (eg, trust in public institutions, solidarity in society, welfare, digitalization of the health sector, and eHealth literacy).

#### Support for Digital Health Data Sharing

Most respondents preferred not to share their health data (84.31%; Table 3). However, some of these respondents may be open to data sharing upon meeting certain conditions, thus showing conditional support. In each region, only a minority showed strong support for data sharing. This reflects the findings of previous empirical studies, which showed that support for health data sharing is not unconditional. Suitable control mechanisms, adequate transparency, and information on data use were common conditions for support identified in different studies [35,36,42]. From a European perspective, measures to create the conditions for trustworthy data-sharing contexts and to establish adequate governance mechanisms for digital health data sharing would be needed to promote citizens’ support for digital health data sharing.

#### Recommendations for a Harmonized Process

As a whole, the preferences expressed by the respondents in this study showed that people care about the fate of their data and want to have control of their data being shared. The heterogeneity of preferences for health data sharing among and within European regions may render harmonization initiatives challenging. To provide for differing preferences and to acknowledge the value given to continuous information and data control, interactive informed consent models that enable individual preferences on the use of data within strong and generalized governance may be valuable as a base for developing uniform processes for data reuse within Europe [43,44]. Such a putative dynamically interactive consent model for digital health data sharing may envision categories of items on which the data subject is called to express a choice, which can be changed over time, thus providing for variations in preferences. For example, data subjects may be offered options on the type of collector, user, and purpose. Instead, an adaptive governance process shared within Europe that allows tailoring to the individual countries’ legislative and regulatory frameworks may define the information that must be provided, the typology of consent (opt-in and opt-out), and the necessary overview mechanisms. This would allow to offer a granularity of choices that adequately address the contextuality of data sharing; to provide meaningful and transparent information that guarantees data subject awareness of the use of the collected data; to provide consent mechanisms that are adequate in relation to the original consent and the contextual use of data; to protect individual rights and autonomy; and to provide oversight mechanisms that guarantee trustworthiness and transparency of the data sharing processes. Among the informed consent models [45,46], dynamic consent may be an apt approach for consent to digital health data sharing in a dynamic context. Dynamic consent is implemented within digital platforms; therefore, it would be suitable for the ongoing and progressive general digitalization of health [43,47,48]. It has been used in various contexts, such as biomedical research, biobanking, and clinical settings [43,49,50]. Based on interactive and ongoing communication with research participants or patients, dynamic consent offers the possibility to revise and change choices over time [43]. It has been reported that the possibility of changing choices over time and regular communication favor trust [49,51] and that granular control over data is desirable [52].

Dynamic consent responds to instances that directly arise from the findings obtained in this study. It offers ongoing information about the use of data and, through an interactive approach, offers the public direct control of data use. Dynamic consent would provide transparency in the ongoing use of data, give effect to the right to information, and provide a process for the control and change of preferences in data use. Dynamic consent may serve the interests of the stakeholders involved in data reuse (data collectors, data users, public, policy makers, etc) because it will allow a combination of transparency and individual control (which is desired by the public) and enable preferences to change over time, and will allow the possibility of uses in a variety of contexts.

The proposed amendments to the draft EHDS introduce an opt-out, thus providing an avenue for the expression of individual preferences. In fact, dynamic consent may be conceived as a possibility for providing information interactively with an opt-out option, thus following the same direction as the proposals in the draft opinion of Parliament on the EHDS.

The dynamic consent model that we propose here relates to the ethical requirement of consent to research as distinct to consent as a legal basis for the processing of personal data under the General Data Protection Regulation (GDPR). However, the dynamic consent model, if modified to have opt-in options only, could also meet the GDPR requirements of consent as a legal basis. This again demonstrates the adaptability of the model. If the infrastructure is put in place, it can be adapted to suit current legal and ethical requirements as they evolve.

While enhancing the legitimacy of data repurposing, there is a risk that dynamic consent measures will privilege the most resourceful citizens who are most likely to have the means for navigating an increasingly complex digital infrastructure. How to balance these concerns will remain a political and moral challenge. Nonetheless, increasing digital literacy and access to digital resources will be key to promote autonomy and fairness.

### Limitations

By design, the sample for each country aimed to reflect the respective national age range and gender distribution of the adult population (as of the most recent official information available from the respective national institutes of statistics at the time of sample design). Our data showed a generally higher proportion of female respondents and slight differences in mean age and educational level among the regions and countries. Previous studies reported that people of different ages had different levels of trust, risk perception, privacy concerns, data sharing attitudes, and willingness to share data [39,40,53,54]. Associations between education level and data sharing attitude, education, and preference for a review of data access were found in previous studies [35,40]. Further analysis aiming to investigate the relationship between the above-described factors and expressed preferences for governance mechanisms would be relevant in understanding and characterizing the variety of preferences in the European population.

This study did not include any Eastern European countries. Due to time and project budget constraints, we could not include additional countries in the study. This is a limitation of the generalizability of the results to Europe. It would be very important to extend the study further and ensure that all the European regions are covered to inform policy accurately and minimize possible biases in the results.

As an expansion of this project, the survey may be distributed in other countries worldwide. This would allow obtaining further insights, which would be valuable to grasp differences and similarities in people’s preferences for the governance of digital health data taking into account geographical regions and contexts of data sharing. In this paper, we decided to group the countries according to the UN classification of European areas. The use of the UN geoscheme would facilitate comparison and generalization according to a shared and globally known scheme in case the study is expanded worldwide. From a conceptual perspective, within the qualitative phase of the project, England, Iceland, and Sweden were grouped because those countries shared “similarities in breaches of trust among the public regarding secondary uses of health data” [42]. Additionally, in the first round of quantitative analysis [15], including the United Kingdom, Iceland, Norway, and Sweden, we implicitly followed the UN scheme; therefore, we decided to repropose the same grouping in this study. All the data were collected through online surveys during the COVID-19 pandemic. It was reported that the pandemic impacted patient preferences for data sharing, resulting in increased comfort in personal health data sharing compared to the prepandemic time [55]. We may speculate that during the pandemic, the growth of digitalization in every aspect of life [56], the role of the internet and media in providing health information, the emergence of digital health technologies (contact tracing apps and approaches for digital medicine) [57-60], and the efforts in data sharing for research and public health purposes [61,62] may have impacted the respondents’ attitudes and views on digital health data sharing and the expressed preferences. As the study was designed since its conception as an online survey, we believe that the findings were not affected by the method of data collection.

### Conclusion

This study, which explored public preferences in 12 European countries, showed the co-existence of overarching priorities (such as the importance of information and consent) and heterogeneous preferences for contexts of data sharing among and within European regions. This study has confirmed the previous findings of a study in Northern European countries [15], provided further nuances to the preferences of Northern European countries, and added the preferences of residents in Western and Southern Europe. It allowed us to understand the pattern of preferences for digital health data sharing in a much broader context and according to geographical regions. With these results, we were able to discuss the challenges of data-sharing harmonization initiatives within Europe. Based on these results, we believe that there is no “one size fits all” governance solution. Instead, an interactive and dynamic model of informed consent offering individual granular control over data sharing accompanied by oversight mechanisms may be a valuable compromise to provide people with the ability to control the secondary use of their health data and to address their preferences for data flow within different contexts. These preferences are contrary to some of the proposals contained within the EHDS. Although the EHDS proposed an independent review of the secondary use of data by a new public entity, a health data access body, the draft regulation on the EHDS removes any role for consent or individual control in the secondary use of data, and it is proposed that individuals will not have a right to be informed about the purpose and the entity that has accessed and used their data. Harmonization initiatives seeking to provide a common ground for cross-border digital health data sharing should be developed upon empirical evidence. Understanding public preferences for digital health data sharing is important for developing adequate answers in policy-making and ensuring that new initiatives are perceived to be trustworthy and operating in accordance with people’s expectations.

## Appendix

Multimedia Appendix 1 Regional preferences.

## Abbreviations

DCE: discrete choice experiment
EHDS: European Health Data Space
EU: European Union
GDPR: General Data Protection Regulation
LCA: latent class analysis
LCM: latent class model
