# Supplementary material for: Public Preferences for Digital Health Data Sharing: Discrete Choice Experiment Study in 12 European Countries
Source: J Med Internet Res. 2023 Nov 23;25:e47066. doi: 10.2196/47066 (PMC10704315; doi:10.2196/47066)
Supplement: Multimedia Appendix 1 [file jmir_v25i1e47066_app1.docx]

## Multimedia Appendix 1. Regional preferences

Tables S1, S2, and S3 show the results of the latent class models of each region. Classes are numbered according to respondents’ *a priori* preference for sharing their health data (see Rejecting data sharing, Intercept), where class 1 is the most negative towards data sharing class 4 is the most positive, and class 2 and 3 showed conditional support or indifference (for a comparison with the outcomes in the main manuscript, class 4 of the regional results matches class 3 of the whole sample results).

### Northern Europe

As shown in Table S1, class 1 respondents were very negative towards data sharing. Class 2 and 3 respondents preferred not to share their digital health data; however, they could become supportive under specific conditions of data sharing that these respondents found preferable (conditional support). Class 4 respondents preferred to accept data sharing.

As data collectors, Northern European respondents of class 1, 2 and 4 preferred their health care provider over an academic research project (not significant for class 1). The opposite was found for class 3 respondents. Technological companies provided disutility in all the classes.

Northern Europe respondents of all classes found it most acceptable to share their health data with a national authority and expressed the most disutility for sharing with a technological company. Respondents in class 2 and 4 showed disutility for sharing their health data with a pharmaceutical company compared with a national authority. Finally, an academic research project was found acceptable for respondents of class 1, 2, and 4 but less than a national authority.

Respondents from Northern Europe in class 2 and 4 preferred to share digital health data for evaluation of quality of the data users’ product or service over development of a new product or service and investigating a policy initiative (not significant in class 4). Class 3 respondents preferred quality evaluation, over investigating a policy initiative and expressed disutility developing a new product or service. Class 1 respondents preferred sharing for the development of a new product or service compared to quality evaluation. Respondents in all classes showed strong disutility for sharing health data for promoting, advertising, or marketing purposes.

In class 1 and 2, respondents preferred being informed and having the ability to opt out compared to being informed with an active consent. The opposite was found for class 3 and 4 respondents, who preferred being informed and asked to consent over having the ability to opt-out. They also expressed disutility for being only informed. In all the classes, being not informed provided the most disutility.

In class 1 and 4, respondents preferred a committee that oversees the transfer only compared to a committee that oversees both the data transfer and use. On the other hand, class 2 respondents preferred the review of data transfer and use over the review of the transfer only. In class 3, the two review levels were equally important. No review mechanisms provided disutility in all the classes.

Compared to respondents from the UK, respondents from Sweden were less likely to belong to class 3 (compared to class 4). Respondents from Norway were more likely to belong to class 4, than any other of the classes. Respondents from Denmark and Iceland were less likely to belong to class 1 and 3. Respondents from Ireland were less likely to belong to class 1.

### Central Europe

Central Europeans’ preferences are shown in Table S2. Class 1 respondents *a priori* strongly preferred not to share their digital health data, while class 4 respondents strongly preferred to accept data sharing. Class 2 respondents preferred not sharing their data but might be conditionally supportive depending on the context. Class 3 respondents did not care.

As data collectors, class 2, 3, and 4 respondents preferred their health care provided over an academic research project (not significant for class 4). Class 1 respondents found most acceptable an academic research project. Respondents of all classes did not prefer a technological company.

Respondents in classes 1, 3, and 4 preferred to share the health data with an academic research project. They expressed disutility for sharing data with a technological company (least acceptable in classes 1 and 3) and with a pharmaceutical company (least acceptable in class 4) compared to a national authority. For respondents in class 2, this attribute did not affect preferences.

Class 1 respondents preferred to share health data to develop a new product or service. Respondents of classes 3 and 4 preferred to share their health data for the evaluation of the quality of a product or service over the development of a new product or service and investigating a policy initiative. Similarly, class 2 respondents preferred to share health data for quality evaluation but expressed disutility for the development of a new product or service. Sharing health data for promotion, advertisement, and marketing purposes provided disutility for all the classes.

Being informed and asked to consent was preferred by all classes over being informed and having the option to opt-out. Being informed only about health data sharing was acceptable for respondents of class 3 (even though less acceptable than being informed and having the option to opt out), while it provided disutility for class 2 respondents. Sharing health data without being informed provided the most disutility for respondents of all classes.

In classes 2, 3, and 4, respondents preferred the review of transfer and use over the review of transfer only. Instead, class 1 respondents preferred the review of transfer only. The absence of review provided disutility in all the classes.

Compared to respondents from the Netherlands, respondents from Germany, France and Austria were less likely to belong to class 3 (compared to class 4). Respondents from the Netherlands were more likely to belong to class 3.

### Southern Europe

Table S3 shows the results of the model in the southern region. Class 1 respondents *a priori* preferred not to share their digital health data. Even though class 2 and 3 respondents *a priori* preferred not to share their data, they might be conditionally supportive given specific conditions. Class 4 respondents preferred to accept data sharing.

As data collectors, while respondents of classes 2, 3, and 4 preferred their health care provider and expressed disutility for a technological company, the opposite pattern was found for respondents of class 1. For class 2 respondents, an academic research project was also acceptable but less than their health care provider.

Class 1 respondents preferred to share digital health data with a pharmaceutical company over a national authority. For them, sharing with an academic research project provided disutility. For respondents in class 2 the data user did not impact respondents’ decision. Respondents of class 3 preferred to share health data with an academic research project and expressed disutility for a technological company. Class 4 respondents preferred to share digital health data with a national authority.

As a reason for data use, for class 1 respondents, investigating a policy initiative provided disutility. Class 2 respondents found most preferrable to share digital health data for quality evaluation while expressed disutility for developing a new product or service and investigating a policy initiative. Class 3 and 4 respondents found the most acceptable sharing data for quality evaluation and least acceptable for promotion, advertising, and marketing. Class 3 respondents also found acceptable sharing for the development of a product or service (but less than quality evaluation) and expressed disutility for investigating a policy initiative compared to quality evaluation.

Class 1 respondents preferred to be only informed about data sharing. Class 2 and 4 respondents preferred to be informed and asked to consent over informed and opt-out. Class 3 respondents preferred being informed and having the option to opt-out. Not being informed provided disutility in all the classes.

Class 1 respondents preferred the absence of review over a review of transfer and use, which provided disutility. On the contrary, for respondents of classes 2, 3 and 4, the absence of review provided the most disutility compared to the presence of review mechanisms. Class 2 and 4 respondents preferred the review of transfer and use over the review of transfer only, while class 3 preferred the review of transfer only.

Compared to respondents from Spain, respondents from Italy were more likely to belong to classes 1 and 3 (compared to class 4). Respondents from Spain were more likely to belong to class 4.

Table S1: Latent class model of Northern Europe respondents’ preferences for digital health data sharing.

| Attribute | Level | Class 1 | | Class 2 | | Class 3 | | Class 4 | |
| --- | --- | --- | --- | --- | --- | --- | --- | --- | --- |
|  |  | Coef. | SE | Coef. | SE | Coef. | SE | Coef. | SE |
| Data collector | Health care provider (Ref) | 0.20^**^ | 0.10 | 0.20^***^ | 0.03 | 0.16^***^ | 0.05 | 0.29^***^ | 0.06 |
|  | Technological company | -0.24^***^ | 0.09 | -0.37^***^ | 0.03 | -0.40^***^ | 0.05 | -0.38^***^ | 0.06 |
|  | Academic research project | 0.04 | 0.09 | 0.16^***^ | 0.03 | 0.23^***^ | 0.04 | 0.09^*^ | 0.05 |
| Data user | National authority (Ref) | 0.27^**^ | 0.12 | 0.32^***^ | 0.04 | 0.10^*^ | 0.05 | 0.37^***^ | 0.07 |
|  | Technological company | -0.39^***^ | 0.14 | -0.49^***^ | 0.04 | -0.12^**^ | 0.06 | -0.53^***^ | 0.07 |
|  | Pharmaceutical company | -0.10 | 0.12 | -0.10^***^ | 0.04 | -0.03 | 0.06 | -0.12^*^ | 0.07 |
|  | Academic research | 0.22^**^ | 0.11 | 0.28^***^ | 0.04 | 0.06 | 0.05 | 0.28^***^ | 0.07 |
| Reason for data use | Quality evaluation (Ref) | 0.28^**^ | 0.12 | 0.48^***^ | 0.04 | 0.36^***^ | 0.06 | 0.46^***^ | 0.07 |
|  | Development of a new product or service | 0.61^***^ | 0.14 | 0.37^***^ | 0.04 | -0.06 | 0.06 | 0.36^***^ | 0.07 |
|  | Promotion, advertising, marketing | -1.00^***^ | 0.16 | -1.06^***^ | 0.05 | -0.46^***^ | 0.07 | -0.85^***^ | 0.08 |
|  | Investigating policy initiative | 0.11 | 0.12 | 0.21^***^ | 0.04 | 0.16^***^ | 0.06 | 0.02 | 0.07 |
| Information | Informed and consent (Ref) | 0.68^***^ | 0.13 | 0.34^***^ | 0.04 | 1.93^***^ | 0.10 | 0.89^***^ | 0.09 |
|  | Not informed | -1.38^***^ | 0.16 | -0.68^***^ | 0.05 | -2.92^***^ | 0.09 | -1.48^***^ | 0.10 |
|  | Informed | -0.08 | 0.14 | -0.02 | 0.04 | -0.52^***^ | 0.06 | -0.13^*^ | 0.07 |
|  | Informed and ability to opt-out | 0.78^***^ | 0.16 | 0.36^***^ | 0.04 | 1.51^***^ | 0.08 | 0.73^***^ | 0.07 |
| Review | Review of transfer and use (Ref) | 0.36^***^ | 0.12 | 0.47^***^ | 0.03 | 0.29^***^ | 0.05 | 0.31^***^ | 0.06 |
|  | No review | -0.89^***^ | 0.11 | -0.91^***^ | 0.04 | -0.58^***^ | 0.06 | -0.77^***^ | 0.07 |
|  | Review of transfer | 0.52^***^ | 0.11 | 0.44^***^ | 0.03 | 0.29^***^ | 0.05 | 0.46^***^ | 0.06 |
| Rejecting data sharing (Intercept) | | 3.52^***^ | 0.12 | 0.30^***^ | 0.04 | 0.82^***^ | 0.05 | -1.92^***^ | 0.07 |
| Class share |  | 24.03% | | 32.63% | | 24.53% | | 18.81 | |
| Class membership variables^a^ | | | | | | | | | |
| Constant | | 0.94^***^ | 0.17 | 0.71^***^ | 0.18 | 0.82^***^ | 0.17 | Ref. | |
| Sweden | | -0.31 | 0.22 | 0.06 | 0.23 | -0.91^***^ | 0.24 |  |  |
| Norway | | -1.03^***^ | 0.22 | -0.72^***^ | 0.24 | -0.51^**^ | 0.22 |  |  |
| Denmark | | -1.51^***^ | 0.24 | -0.34 | 0.23 | -1.03^***^ | 0.23 |  | |
| Iceland | | -0.75^***^ | 0.22 | 0.10 | 0.22 | -0.94^***^ | 0.23 |  | |
| Ireland | | -0.67^***^ | 0.25 | -0.11 | 0.26 | 0.18 | 0.24 |  | |

***, **, *Significant at 1%, 5%, 10% level; ^a^Reference class membership: United Kingdom (UK)

Table S2: Latent class model of Central Europe respondents’ preference for digital health data sharing.

| Attribute | Level | Class 1 | | Class 2 | | Class 3 | | Class 4 | |
| --- | --- | --- | --- | --- | --- | --- | --- | --- | --- |
|  |  | Coef. | SE | Coef. | SE | Coef. | SE | Coef. | SE |
| Data collector | Health care provider (Ref) | 0.02 | 0.12 | 0.16^***^ | 0.07 | 0.22^***^ | 0.03 | 0.48^***^ | 0.11 |
|  | Technological company | -0.22^*^ | 0.12 | -0.31^***^ | 0.06 | -0.28^***^ | 0.03 | -0.52^***^ | 0.13 |
|  | Academic research project | 0.20^*^ | 0.11 | 0.15^***^ | 0.06 | 0.06^*^ | 0.03 | 0.05 | 0.12 |
| Data user | National authority (Ref) | 0.21 | 0.14 | -0.09 | 0.07 | -0.03 | 0.04 | 0.26 | 0.17 |
|  | Technological company | -0.46^***^ | 0.15 | -0.03 | 0.08 | -0.17^***^ | 0.04 | -0.27^**^ | 0.13 |
|  | Pharmaceutical company | -0.13 | 0.15 | 0.07 | 0.08 | -0.01 | 0.04 | -0.60^***^ | 0.17 |
|  | Academic research project | 0.38^**^ | 0.15 | 0.05 | 0.08 | 0.20^***^ | 0.04 | 0.61^***^ | 0.16 |
| Reason for data use | Quality evaluation (Ref) | 0.12 | 0.14 | 0.57^***^ | 0.10 | 0.31^***^ | 0.04 | 0.56^***^ | 0.15 |
|  | Development of a new product or service | 0.44^***^ | 0.16 | -0.21^***^ | 0.07 | 0.21^***^ | 0.04 | 0.27^*^ | 0.14 |
|  | Promotion, advertising, marketing | -0.40^**^ | 0.18 | -0.47^***^ | 0.10 | -0.68^***^ | 0.05 | -1.07^***^ | 0.18 |
|  | Investigating policy initiative | -0.16 | 0.14 | 0.12 | 0.08 | 0.16^***^ | 0.04 | 0.25^*^ | 0.13 |
| Information | Informed and consent (Ref) | 0.76^***^ | 0.18 | 1.82^***^ | 0.11 | 0.33^***^ | 0.05 | 1.41^***^ | 0.21 |
|  | Not informed | -1.38^***^ | 0.17 | -3.04^***^ | 0.14 | -0.67^***^ | 0.06 | -1.82^***^ | 0.27 |
|  | Informed | 0.25 | 0.18 | -0.25^***^ | 0.08 | 0.10^**^ | 0.04 | 0.00 | 0.15 |
|  | Informed and ability to opt-out | 0.37^**^ | 0.17 | 1.46^***^ | 0.11 | 0.24^***^ | 0.04 | 0.42^**^ | 0.17 |
| Review | Review of transfer and use (Ref) | 0.18 | 0.12 | 0.65^***^ | 0.09 | 0.17^***^ | 0.04 | 0.41^***^ | 0.12 |
|  | No review | -0.68^***^ | 0.13 | -1.21^***^ | 0.09 | -0.30^***^ | 0.05 | -0.71^***^ | 0.13 |
|  | Review of transfer | 0.51^***^ | 0.13 | 0.56^***^ | 0.07 | 0.13^***^ | 0.04 | 0.30^***^ | 0.12 |
| Rejecting data sharing (Intercept) | | 2.88^***^ | 0.16 | 0.56^***^ | 0.08 | -0.02 | 0.04 | -2.97^***^ | 0.17 |
| Class share |  | 18.51% | | 24.57% | | 40.44% | | 16.48% | |
| Class membership variables^a^ | | | | | | | | | |
| Constant | | 0.45 | 0.36 | 0.31 | 0.38 | 2.49^***^ | 0.27 | Ref | |
| Germany | | -0.49 | 0.37 | 0.15 | 0.39 | -2.20^***^ | 0.31 |  |  |
| France | | -0.52 | 0.37 | -0.10 | 0.40 | -2.17^***^ | 0.30 |  |  |
| Austria | | -0.31 | 0.37 | 0.31 | 0.40 | -1.84^***^ | 0.31 |  |  |

***, **, * Significant at 1%, 5%, 10% level; ^a^Reference class membership: Netherlands

Table S3: Latent class model of Southern Europe respondents’ preference for digital health data sharing.

| Attribute | Level | Class 1 | | Class 2 | | Class 3 | | Class 4 | |
| --- | --- | --- | --- | --- | --- | --- | --- | --- | --- |
|  |  | Coef. | SE | Coef. | SE | Coef. | SE | Coef. | SE |
| Data collector | Health care provider (Ref) | -0.72^**^ | 0.31 | 0.33^***^ | 0.11 | 0.15^***^ | 0.05 | 0.35^***^ | 0.10 |
|  | Technological company | 1.10^***^ | 0.35 | -0.63^***^ | 0.11 | -0.22^***^ | 0.05 | -0.38^***^ | 0.11 |
|  | Academic research project | -0.38 | 0.28 | 0.30^***^ | 0.11 | 0.07 | 0.05 | 0.03 | 0.11 |
| Data user | National authority (Ref) | -0.71^**^ | 0.33 | -0.08 | 0.11 | 0.08 | 0.06 | 0.25^*^ | 0.14 |
|  | Technological company | -0.07 | 0.41 | 0.06 | 0.15 | -0.25^***^ | 0.07 | -0.05 | 0.12 |
|  | Pharmaceutical company | 1.62^**^ | 0.63 | 0.09 | 0.14 | 0.03 | 0.07 | -0.23 | 0.14 |
|  | Academic research project | -0.84^**^ | 0.38 | -0.06 | 0.12 | 0.15^**^ | 0.07 | 0.03 | 0.15 |
| Reason for data use | Quality evaluation (Ref) | -0.65 | 0.72 | 0.73^***^ | 0.20 | 0.40^***^ | 0.07 | 0.46^***^ | 0.14 |
|  | Development of a new product or service | -0.14 | 0.68 | -0.22^*^ | 0.12 | 0.27^***^ | 0.07 | -0.09 | 0.14 |
|  | Promotion, advertising, marketing | 2.50 | 1.93 | -0.27 | 0.17 | -0.40^***^ | 0.08 | -0.29^**^ | 0.15 |
|  | Investigating policy initiative | -1.71^**^ | 0.70 | -0.24^*^ | 0.14 | -0.27^***^ | 0.07 | -0.07 | 0.14 |
| Information | Informed and consent (Ref) | -0.49 | 0.57 | 2.06^***^ | 0.25 | 0.11 | 0.08 | 0.98^***^ | 0.17 |
|  | Not informed | -1.31^***^ | 0.29 | -3.52^***^ | 0.29 | -0.43^***^ | 0.09 | -1.43^***^ | 0.21 |
|  | Informed | 1.79^***^ | 0.49 | -0.12 | 0.13 | 0.10 | 0.07 | 0.02 | 0.14 |
|  | Informed and ability to opt-out | 0.01 | 0.57 | 1.58^***^ | 0.17 | 0.22^***^ | 0.07 | 0.42^***^ | 0.14 |
| Review | Review of transfer and use (Ref) | -0.80^***^ | 0.29 | 0.48^***^ | 0.15 | 0.09 | 0.06 | 0.27^**^ | 0.11 |
|  | No review | 1.06^***^ | 0.34 | -0.71^***^ | 0.16 | -0.21^***^ | 0.06 | -0.46^***^ | 0.12 |
|  | Review of transfer | -0.26 | 0.36 | 0.23^**^ | 0.11 | 0.13^**^ | 0.06 | 0.19^*^ | 0.11 |
| Rejecting data sharing (Intercept) | | 4.69^***^ | 0.76 | 0.86^***^ | 0.10 | 0.63^***^ | 0.05 | -1.40^***^ | 0.12 |
| Class share |  | 25.27% | | 26.11% | | 35.12% | | 13.51% | |
| Class membership variables^a^ | | | | | | | | | |
| Constant | | 0.37^**^ | 0.18 | 0.47^***^ | 0.18 | 0.19 | 0.20 | Ref | |
| Italy | | 0.54^*^ | 0.30 | 0.39 | 0.32 | 1.62^***^ | 0.30 |  |  |

***, **, * Significant at 1%, 5%, 10% level; ^a^Reference class membership: Spain
